# The Role of HPV and Non-HPV Sexually Transmitted Infections in Patients with Oropharyngeal Carcinoma: A Case Control Study

**DOI:** 10.3390/cancers12051192

**Published:** 2020-05-08

**Authors:** Barbara Kofler, Johannes Laimer, Emanuel Bruckmoser, Teresa B. Steinbichler, Annette Runge, Volker H. Schartinger, Dorothee von Laer, Wegene Borena

**Affiliations:** 1 Department of Otorhinolaryngology, Medical University of Innsbruck, Anichstrasse 35, 6020 Innsbruck, Austria; ba.kofler@tirol-kliniken.at (B.K.); teresa.steinbichler@i-med.ac.at (T.B.S.); annette.runge@tirol-kliniken.at (A.R.); volker.schartinger@i-med.ac.at (V.H.S.); 2 University Hospital of Cranio-Maxillofacial and Oral Surgery, Medical University of Innsbruck, 6020 Innsbruck, Austria; johannes.laimer@i-med.ac.at; 3 Independent Researcher, 5020 Salzburg, Austria; research@bruckmoser.info; 4 Institute of Virology, Department of Hygiene, Microbiology, Social Medicine, Medical University of Innsbruck, Peter-Mayr-Strasse 4b, 6020 Innsbruck, Austria; dorothee.von-Laer@i-med.ac.at

**Keywords:** Ureaplasma spp., sexually transmitted infections, human papillomavirus, oropharyngeal cancer, brush test

## Abstract

*Background*: Certain high-risk (hr) types of human papillomavirus (HPV) can cause cervical cancer in women and penile cancer in men. Hr-HPV can also cause cancers of the oropharynx and anus in both sexes. In the anal and cervical region, a contribution of co-infections with Ureaplasma spp. on the persistence of the hr-HPV infection by a profound inflammatory state is suggested. Here, we investigated if non-HPV sexually transmitted infections are associated with oropharyngeal carcinoma (OPC). *Materials and Methods*: In this case-control study, a brush test directly from the tumor surface of OPC patients (study group) and from the oropharynx of healthy volunteers (control group), both groups matching in age and sex, was performed. HPV subtypes were detected using a commercially available test kit. For non-HPV sexually transmitted infections (Ureaplasma spp., Chlamydia trachomatis, Mycoplasma hominis, and Mycoplasma genitalium), a multiplex nucleic acid amplification approach was performed. *Results*: In the study group, 96 patients (23 female/73 male), with histologically confirmed OPC and in the control group 112 patients (19 female/93 male), were included. Oropharyngeal hr-HPV-positivity was detected in 68% (65/96 patients) of the study group and 1.8% (2/112 patients) of the control group (*p* < 0.001). In three patients in the study group, Ureaplasma spp. was detected, whereas no patient was Ureaplasma spp. positive in the control group (*p* = 0.097). Chlamydia trachomatis, Mycoplasma hominis, and Mycoplasma genitalium were negative in both groups. *Conclusion*: Based on the current study, the prevalence of oropharyngeal Ureaplasma spp. among patients with OPC is low and does not support a role in oropharyngeal cancer. However, the detection of the pathogen only among OPC patients but not in the healthy individuals might indicate a potential role and needs further elucidation.

## 1. Introduction

Changes in sexual behavior in the last decades, like a high number of oral sex partners, seem to play an important etiological role in the development of human papillomavirus (HPV) positive oropharyngeal carcinoma (OPC) by HPV transmission [1]. Once an oropharyngeal HPV infection has occurred, the infection is usually asymptomatic and eliminated by the immune system within a few months. Only a small proportion of these infections persist and progress to dysplastic lesions or cancer, and only the persistence of the virus is oncogenic [2].

Therefore, it is of high importance to understand which factors and mechanisms may contribute to the persistence of an oropharyngeal HPV-infection and lead to the development of OPC.

Co-factors like smoking and immunosuppression were reported to play a meaningful role in the process of HPV-persistence [3,4]. Studies in the past reported that a co-existence of HPV with sexually transmitted infections (STIs) facilitates the persistence and dysplastic transformations of HPV-associated lesions in the cervix or the anus [5,6,7,8,9,10,11,12,13,14].

Mycoplasma and Ureaplasma, a distinguished form of bacteria characterized by their minute size and total lack of a cell wall, were reported to play an important role in HPV infections, abnormal cervical cytopathology, and cervical cancer. Ureaplasma urealyticum and Mycoplasma genitalium may increase the risk of a high-risk (hr) HPV infection, while Ureaplasma urealyticum, Ureaplasma parvum, and Mycoplasma hominis may increase the risk of abnormal cervical cytopathology [15].

The bacteria are widespread in nature and well known as human parasites, often causing chronic asymptomatic infections. Mycoplasmas usually exhibit a rather strict host and tissue specificity, and the primary habitats are the mucous surfaces of the respiratory and urogenital tracts, the eyes, alimentary canal, mammary glands, and joints [16]. Because mycoplasmas possess the smallest genome known for free-living organisms, the autonomy of the bacteria is limited and makes them susceptible to changes in the host organism. Many mycoplasmas themselves cause pathological changes in the host organism, often complicated by immune disorders. Furthermore, mycoplasmas can inhibit the p53-mediated checkpoint control of the cell cycle and apoptosis. This indicates that mycoplasmas might act as a cancer-promoting factors [17]. In vitro studies described the potential of a Mycoplasma species to a malignant transformation and chromosomal instability in long term Mycoplasma infected cell cultures [18,19,20]. Moreover, in epidemiological studies, the detection of Mycoplasma strains in cancer samples or antibodies against these microorganisms in cancer patients has been documented [21]. Idhal and coworkers evaluated the presence of anti-M genitalium antibody in 291 women with ovarian cancer. Their results were suggestive of a potential association between M genitalium and ovarian cancer (*p* = 0.01) [22]. Barykova and coworkers described M. hominis to be three times more frequent in patients with prostate cancer than in those with benign prostatic hyperplasia and suggested that M. hominis infections may be involved in prostate cancer development [23]. Mizuki and coworkers described a correlation between oral leukoplakia and M. salivarium using an immunohistochemical analysis. The authors observed small granular fluorescing structures in the cytoplasm of oral leukoplakia cells, which they identified, based on its morphology and size, as Mycoplasma species [24]. The substantial increase in the presence of Mycoplasma within the cytoplasm of oral leukoplakia as compared to the control group with normal oral mucosa cells further strengthened the potential role of such bacterial pathogen in the development of malignant lesions [25]. Other common sexually transmitted pathogens like C. trachomatis are considered to be possible co-factors facilitating HPV associated oncogenesis. An association between a C. trachomatis infection and cervical cancer or its precursor lesions has been described in several previous studies. Potential mechanisms include alteration of the epithelial tissue due to local inflammatory response making the region susceptible to HPV infection [26,27,28].

Colleagues of our study group collected recent anal brush samples in 222 HIV-positive men who have sex with men (MSM) for the detection of Chlamydia trachomatis, Neisseria gonorrhea, Ureaplasma spp., Mycoplasma, and HPV genotypes. Out of these participants, 73% were hr HPV-positive, and 19.4% harbored Ureaplasma spp. Hr HPV-infection was significantly associated with the co-presence of Ureaplasma spp. (OR 2.59, 95% CI: 1.03–6.54) [14].

Given the similar routes of transmission, the co-presence of HPV with other STIs is not a surprise even in the oropharyngeal region. Although previous studies reported on the frequency of Mycoplasma and Ureaplasma spp. detection in the oral cavity [29], data on HPV and non-HPV sexually transmitted infections (STI) are inexistent among patients with OPC.

The aim of the study was to evaluate the prevalence of HPV and non-HPV sexually transmitted infections among patients with a pathologically confirmed malignant tumor of the oropharynx. To further validate the results, outcomes were compared to a control group of volunteers without head and neck squamous cell carcinoma (HNSCC). The null hypothesis was that there is no difference in the prevalence of non-HPV sexually transmitted infections among patients with and without OPC.

## 2. Results

### 2.1. Study Population

A total of 208 adult men and women (*n*_OPC_ = 96 and *n*_healthy_ = 112) were included. The mean age (SD) of the study participants was 61.9 (10.1) years. There was no significant difference in the age distribution between OPC patients and healthy controls, 61.9 (9.7) versus 62.1 (10.5) years, respectively. In the study group, the proportion of female patients (23.9%) was slightly higher than in the control group (17%) (Table 1). Further clincal-pathological parameters of the study group revealed that 68% of the OPC patients were HPV-positive, radiochemotherapy was the most common treatment modality, and most patients were classified as UICC stage IV. HPV-positive OPC patients were obviously younger than the control group (*p* = 0.006), had lower T-stage (*p* < 0.001), and lower ASA scores (*p* < 0.001). P16 was in 88.5% of the HPV-associated OPC patients positive (*p* < 0.001) (Table 2).

### 2.2. HPV-DNA Detection

A total of 65 (67%) of the OPC patients were positive for HPV DNA. The most common genotype was HPV 16 (n_OPC_ = 52), followed by HPV 18 (n_OPC_ =4), HPV 33 (n_OPC_ =3) and HPV 35 (n_OPC_ =3). Moreover, the HPV types HPV 31, HPV 40, HPV 56, HPV 58, HPV 61, HPV 62, HPV 66, HPV 70, HPV 73 and HPV 82 were detected as a single infection or additionally to HPV 16 as a multiple infection (Table 3). In the control group, two individuals (1.8%) harbored low-risk HPV DNA in the oropharynx. The detected HPV types were HPV 70 and HPV 64 (Table 3).

### 2.3. Detection of Non-HPV Sexually Transmitted Infections

Three OPC patients (3.1%) were tested positive for Ureaplasma spp., which was detected in two patients as a co-infection with HPV 16, and in one patient with an HPV-negative OPC. All other non-HPV sexually transmitted infections (Chlamydia trachomatis, Mycoplasma hominis, and Mycoplasma genitalium) were negative in the study group. In the control group, all investigated non-HPV sexually transmitted infections were tested negative. The difference between the frequency of the Ureaplasma spp. infections in the study group (three patients) versus the control group (zero patients) did not reach statistical significance (*p* = 0.097) (Table 1).

## 3. Discussion

To the best of our knowledge, this is the first study to examine the presence of HPV in combination with non-HPV STIs in oropharyngeal samples of patients with histologically confirmed OPCs.

### 3.1. HPV and Oropharyngeal Carcinoma

In our study, we found that the prevalence of HPV-positivity was high (68%) in the 96 OPC patients. HPV-positive OPC patients were younger and had a lower ASA score, which indicates a lower rate of comorbidities [30] as compared to HPV-negative OPC patients. This is in line with other studies, which described how HPV positive patients tend to be young and otherwise healthy [31,32,33]. The most common genotype detected in our study group was HPV 16 (52 patients, 80%), followed by HPV 18 (four patients, 6.2%), HPV 33 (three patients, 4.6%) and HPV 35 (three patients, 4.6%). This is in line with previous investigations. Moreover, Fossum and coworkers reported on 166 OPC patients HPV 16 to be the predominating genotype in 65% of the patients, followed by HPV 33 (17%), HPV 18 (2%), and HPV 31/35/56/59 in one patient each [34].

In the control group, only two patients (1.8%) were HPV-positive. The lack of hr HPV among the control group in our study may be of high diagnostic value. Although hr HPV negativity may not exclude the presence of OPC, based on our study, older adults with hr HPV in the oropharynx may be suspicious of having a head and neck malignancy. These patients may probably be referred to the department of otorhinolaryngology for clinical examination. There is currently no evidence-based implementation of an OPC screening [35]. Additional studies are necessary in order to establish an OPC risk algorithm and to implement this approach in the associated guidelines.

### 3.2. Non-HPV STI and Oropharyngeal Carcinoma

The use of a multiplex nucleic acid amplification approach enabled the search for the co-existence of bacterial infections, namely, Chlamydia trachomatis, Ureaplasma spp., Mycoplasma hominis, and Mycoplasma genitalium, out of a single brush sample. We used a highly sensitive and well-validated extraction and amplification assays, which make the finding in this study a robust one. Our data show that Ureaplasma spp. was detected in two HPV-positive OPC patients and in one HPV-negative OPC patient. Unfortunately, this low prevalence rate of Ureaplasma spp. makes it impossible to assess the potential role of non-HPV STI in HPV associated malignancy in the oropharynx. With only three OPC patients in the study group and none of the control group testing positive for Ureaplasma spp., as well as none testing positive for the other bacterial pathogens, the prevalence of non-HPV STIs was low in our study (*p* = 0.097).

There is substantial molecular evidence that a non-HPV STI co-infection might be associated with cervical or anal carcinogenesis through the induction of a profound inflammatory state [36,37,38,39]. Drago and coworkers detected a cervical HPV infection in 31 (68%) and a Ureaplasma parvum (UP) infection in 21 (46%) out of 64 women. Eighteen patients positive for UP were co-infected with HPV (86%), of which only two patients (11%) had a normal cytology, whereas 16 (89%) had an abnormal cytology, showing a cervical intraepithelial neoplasia. The authors suggested that UP may be involved in HPV persistence and promotes the development of cytological abnormalities [40]. Moreover, Biernat-Sudolska and coworkers described in 387 women an association between the presence of urogenital mycoplasmas and HPV infections in cervical smears of women diagnosed with abnormal cervical cytology. Their statistical analysis demonstrated that the risk of an HPV co-infection increased two-fold with a concomitant Mycoplasma infection and 4.7-fold with a concomitant Ureaplasma urealyticum infection [12].

Given the increasing incidence of HPV-associated OPC [41], the observed Ureaplasma spp. positivity exclusively among OPC patients in our study may be a non-negligible finding, particularly considering the fact that previous studies had found an association between Ureaplasma positivity and anogenital dysplasias [14].

There is very scarce literature on non-HPV STIs in the oropharynx. Naksahima and coworkers investigated the prevalence of STIs in oral gargles in 213 men without OPC attending the sexually transmitted disease (STD) clinic. The authors detected HPV in 18.8%, N. gonorrhoeae in 15.6%, C. trachomatis in 4.2%, M. genitalium in 5.2%, and Ureaplasma spp. in 16.0% of the oral samples [29]. In contrast, Deguchi and coworkers described a low prevalence of genital mycoplasmas and ureaplasmas in the pharynges of Japanese female sex workers. The prevalence in the pharynx of N. gonorrhoeae, C. trachomatis, M. genitalium, M. hominis, U. parvum, and U. urealyticum was 4.0%, 2.0%, 0%, 1.2%, 0.2%, and 0.7%, respectively [42]. Moreover, a Dutch study which aimed to assess spontaneous clearance of chlamydia among high-risk female patients and men who have sex with men found a relatively low prevalence of pharyngeal chlamydia [43].

Although a previous study went further to analyze the role of non-HPV STI in oral swab samples among patients with oral dysplastic lesions, similar data among OPC patients is nonexistent. Mosmann and coworkers examined benign oral lesions or potentially malignant disorders, including oral cancer patients. They found a prevalence of 34% for HPV, 16% for C. trachomatis, and 3% for HSV. They failed to test for Ureaplasma and Mycoplasma spp. [44]. By going further into examining OPC patients, we believe that our study contributes to the pool of data known to date. The search for not only HPV but also for several other non-HPV STIs among these patients makes this prevalence study a useful addition to the current understanding.

Epidemiological surveys confirm that oral sexual practice significantly declines with increasing age [45,46]. Since the median age of our study population was about 60 years, and clearly older than study participants from previous studies, the low prevalence of non-HPV STIs in the oropharyngeal region in this study may be partly explained by this epidemiological factor. The fact that our study participants come from the department of otorhinolaryngology, head and neck surgery, and not from an STD clinic might be an important factor playing a role regarding the low prevalence of oropharyngeal STIs. Furthermore, we used a pharyngeal brush with proven high sensitivity and specificity for HPV detection on OPC patients [47] and not an oral gargle solution. With this test method, we were able to collect samples precisely from the tumor surface of the oropharyngeal cancer. In contrast to the gargle solution, with the tumor surface brushing approach, there is no further contact with other regions of the oropharynx or the oral cavity. A further strength of the study is the fact that the oropharyngeal brush was exclusively performed by otorhinolaryngologists in the study group and by maxillofacial surgeons in the control group.

Prospective studies need to elaborate on the clinical and epidemiological significance of this colonization. If other studies confirm a differential predominance of Ureaplasma spp. among patients with OPC, the issue of early detection and appropriate antibiotic therapy or eradication might be a topic. Due to the low prevalence, there was no clear association between Ureaplasma spp. with HPV-positivity among these patients in our study.

## 4. Materials and Methods

In this prospective case-control study, consecutive patients scheduled for oropharyngeal tumor diagnosis at the outpatient unit of the Department of Otorhinolaryngology, Medical University Innsbruck, Austria, were invited to participate. The admission period was between May 2014 and April 2019. The control group of healthy volunteers was recruited from patients presenting for dentoalveolar procedures or control examinations at the outpatient clinic of Oral and Maxillofacial Surgery, Medical University Innsbruck, Austria, between October and December 2019.

This project was conducted in accordance with STROBE guidelines for observational studies [48]. Written informed consent was obtained from each patient who agreed to participate in this study following a detailed explanation of the procedural workflow. Prior to any patient enrolment, the study had been approved by the institutional board in charge, i.e., the ethics committee of the Medical University Innsbruck, Austria. The respective reference number was 1147/2018. The study was conducted in full accordance with the principles expressed in the declaration of Helsinki.

Inclusion criteria for participation in the study group comprised of a minimum age of at least 18 years and a histologically confirmed OPC. For the control group, healthy volunteers with an age of over 45 years and without previous or current HNSCC were included.

### 4.1. Sample Collection

For sample collection, a validated brush test was used as described in a previous publication [47]. For OPC patients, tumor surface lesions were brushed several times using a cytology brush (digene^®^ HC2 DNA Collection Device, Qiagen, Hilden, Germany) and rinsed in digene Specimen Transport Medium. Particular attention was paid to perform the brush precisely from the tumor surface without the brush coming into contact with the mucosa of the oral cavity. Oropharyngeal brush samples from control patients were collected by the same method.

### 4.2. Nucleic Acid Extraction

Nucleic acid extraction was conducted in a fully automated manner (NucliSens^®^ easyMAG^®^, Biomerieux, Marcy-l’Étoile, France) according to the manufacturer’s instructions. In brief, out of 500 µL of the original sample, 110 µL of purified nucleic acid was extracted using magnetic silica particles. This method is a highly sensitive approach since it allows the capture of all nucleic acid available in a sample, and the separation from silica leads to no loss of extracted material.

### 4.3. HPV DNA Detection and Genotyping

After real-time amplification of the HPV-L1 genome using a one single-step PCR (5 μL of purified nucleic acid), genotyping followed using allele-specific reverse line-blot hybridization of the PCR products. These biotinylated PCR products then bind with target-specific probes that are bound to a nylon membrane (strip), permitting the differentiation of 40 high-risk (hr-HPV) and low-risk HPV genotypes. HPV genotypes detectable in this assay include types 6, 11, 16, 18, 26, 31, 33, 35, 39, 40, 42, 43, 44, 45, 51, 52, 53, 54, 55, 56, 58, 59, 61, 62, 64, 66, 67, 68a/b, 69, 70, 71, 72, 73, 81, 82, 83, 84, 87, 89, and 90. Beta-globin was used as an internal control assuring the validity of the test by controlling for sample quality, the performance of the extraction, and the validity of nucleic acid amplification. Moreover, the presence of the dUTP/UNG system within the reagents of the kit help prevent carry-over contaminations. (Ampliquality Type Express, AB ANALITICA, Padua, Italy).

### 4.4. Detection of Non-HPV STIs

Ten µl of DNA extract (NucliSens^®^ easyMAG^®^, Biomerieux) was used for the amplification of Ureaplasma spp., Mycoplasma genitalium, Mycoplasma hominis, and Chlamydia trachomatis DNA using a validated multiplex nucleic acid amplification kit (AmpliSens^®^ multiprime-FRT, Moscow, Russia) according to the thermocycling conditions recommended by the manufacturer. The kit is equipped with negative and positive controls, as well as an internal control system verifying the extraction and the amplification steps.

### 4.5. Data Analysis

Data were analyzed using SPSS^®^ (IBM Corp. Released 2016. IBM SPSS Statistics for Windows, Version 24.0. Armonk, NY, USA). Frequency data were tabulated and analyzed with Fisher’s exact test or with the Kruskal-Wallis test for ordered alternatives. No statistics were computed for Chlamydia trachomatis, Mycoplasma hominis, and Mycoplasma genitalium because these are constants in both groups [49]. For continuous data, means and standard deviations (SD) are provided unless stated otherwise [50]. A *p*-value of < 0.05 was considered statistically significant.

## 5. Conclusions

Our study shows that the prevalence of non-HPV sexually transmitted infections among patients with OPC is low. Although Ureaplasma spp. has been implicated as a potential HPV-associated carcinogen in anogenital cancers, the low frequency in this study (both in the HPV-positive and HPV-negative patients) does not support a role in oropharyngeal cancer. However, beyond the obvious predominance of hr-HPV, the detection of Ureaplasma spp. only among OPC patients but not in the age and sex-matched control group represents an interesting finding. Given the fact that Ureaplasma spp. were previously shown to be associated with hr-HPV infection or HPV-associated dysplasia, further evaluation of a potential link between a HPV and Ureaplasma spp. co-infection in the pathogenesis of oropharyngeal carcinoma should be evaluated in larger studies.

## Figures and Tables

**Table 1:** Study population.

Variables	Study Group *n* = 96	Control Group *n* = 112	*p*-Value
**Diagnosis**	OPC	Healthy volunteers	
**Mean age at diagnosis**	61.9 years	62.1 years	
**Sex**	23 female (23.9%) 73 male (76.1%)	19 female (17%) 93 male (83%)	
**HPV-positivity**	65 patients (68%)	2 patients (1.8%)	*p* < 0.001
**Ureaplasma spp.**	3 patients (3.1%)	0 patients	*p* = 0.097

OPC, oropharyngeal cancer; HPV, human papillomavirus

**Table 2:** Clinical characteristics of the OPC patients.

Variables	HPV-Positive OSC (*n* = 65)	HPV-Negative OSC (*n* = 31)	*p*-Value
**Sex**			
Male	48 (73.8%)	26 (83.9%)	*p* = 0.204
Female	17 (26.2%)	5 (16.1%)	
**Age**			
≤65 years	48	14	*p* = 0.006
>65 years	17	17	
**Clinical T-stage**			
cT1/T2	48	7	*p* < 0.001
cT3/4	17	24	
**UICCC**			
Stage I	0	2 (6.5%)	*p* = 0.143
Stage II	3 (4.6%)	0	
Stage III	17 (26.2%)	4 (12.9%)	
Stage IV	45 (69.2%)	25 (80.6%)	
**Therapy**			
Surgery only	4 (6.3%)	5 (17.9%)	*p* = 0.183
Surgery and PORT	13 (20.6%)	6 (21.4%)	
Surgery and RCT/RIT	6 (9.5%)	3 (10.7%)	
Primary RCT/RIT	38 (60.3%)	11 (39.3%)	
Primary RT	2 (3.2%)	3 (10.7%)	
**p16**			
Positive	54 (88.5%)	3 (12%)	*p* < 0.001
Negative	7 (11.5%)	22 (88%)	
**ASA score**			
ASA I/II	55 (87.3%)	13 (50%)	*p* < 0.001
ASA III/IV	8 (12.7%)	13 (50%)	

OPC, oropharyngeal cancer; *UICC*, Union for International Cancer Control; *PORT*, postoperative radiation; RCT, radiochemotherapy; RIT, radioimmunotherapy; RT, radiotherapy; *ASA*, American Society of Anesthesiologist.

**Table 3:** HPV subtypes in OPC patients.

HPV Subtypes	Number and Percent of HPV+ OPC Patients
HPV 16 *	52 patients (80%)
HPV 18 *	4 patients (6.2%)
HPV 33 *	3 patients (4.6%)
HPV 35 *	3 patients (4.6%)
HPV 31 *, HPV 40 **, HPV 56 *, HPV 58 *, HPV 61 **, HPV 62 *, HPV 66 *, HPV 70 **, HPV 73 *, HPV 82 *	each in 1 patient or additionally to HPV 16 as multiple infection

HPV, Human Papillomavirus; OPC, oropharyngeal cancer; * high-risk (hr) and probably hr-HPV infection; ** low-risk HPV infection
